# Fracture Resistance of Root-Canal Treated Premolars Restored with Dentin Replacement Materials: An In-vitro Study

**DOI:** 10.3290/j.ohpd.b3170031

**Published:** 2022-06-28

**Authors:** Alhanouf S. Aldegheishem, Reem M. Barakat, Alanood M. AlRabiah, Asmaa H. Binhumaid, Elzahraa Eldwakhly

**Affiliations:** a Associate Professor in Prosthodontics, Clinical Dental Sciences Department, College of Dentistry, Princess Nourah Bint Abdulrahman University, Riyadh, Saudi Arabia. Study concept and design.; b Assistant Consultant in Endodontics. Clinical Dental Sciences Department, College of Dentistry, Princess Nourah Bint Abdulrahman University, Riyadh, Saudi Arabia. Study concept and design, drafted the manuscript, critically revised the manuscript for important intellectual content.; c Dental Intern, College of Dentistry, Princess Nourah Bint Abdulrahman University, Riyadh, Saudi Arabia. Data acquisition.; d Dental Intern, College of Dentistry, Princess Nourah Bint Abdulrahman University, Riyadh, Saudi Arabia. Data acquisition, drafted the manuscript.; e Professor in Prosthodontics, Department of Clinical Dental Sciences, College of Dentistry, Princess Nourah bint Abdulrahman University, Riyadh, Saudi Arabia. revised the manuscript critically for important intellectual content.

**Keywords:** Biodentine, endodontically treated teeth, fracture resistance, restoration, Smart dentin replacement

## Abstract

**Purpose:**

Conservative restorations of endodontically treated premolars have yielded mixed results. The present study aimed to compare fracture resistance of endodontically treated premolars with Class II mesial-occlusal cavity preparations, restored with either Smart Dentin Replacement (SDR; Dentsply Sirona) material, Biodentine (Septodont) or ceramic inlays.

**Materials and Methods:**

Thirty-two extracted premolars were randomly divided into four equal groups (n = 8): Group 1 served as a control group with teeth left intact; teeth in the remaining three groups received root canal treatment followed by a mesio-occlusal cavity preparation. These crowns were restored with: Biodentine in group 2, SDR in group 3 and ceramic inlays in group 4. A computer-controlled Instron universal testing machine subjected all specimens to compressive load until failure. Force at failure and fracture mode (above or below the cementoenamel junction) were recorded. The data were analysed using Fisher’s exact test and one-way ANOVA followed by the post-hoc Tukey’s test. Statistical significance was set at p < 0.05.

**Results:**

The lowest mean load at failure was recorded for the inlay group. Loads at failure were statistically significantly higher for teeth restored with Biodentine than with SDR (p = 0.012) and ceramic inlays (p = 0.007). There were no statistically significant differences between the groups in terms of fracture mode (p = 0.440).

**Conclusion:**

Endodontically treated premolars with mesial-occlusal cavity preparation restored with Biodentine were more resistant to fracture than those restored with either SDR or ceramic inlays. Biodentine may prove a promising material to restore endodontically treated teeth with one missing proximal wall.

Coronal restoration quality is paramount for the long-term success of endodontically treated teeth (ETT).^26^ ETT exhibit increased weakness due to the reduced amount of remaining tooth structure, rendering them more prone to fracture.^11^ Many factors influence the fracture resistance of ETT: tooth type, shape and dimensions of the tooth cavity, in addition to the material used for its restoration.^3,24^ Much controversy exists regarding the types of restoration,^2,14^ used to restore ETT, especially with the advent of adhesive techniques.

Conservative restorative approaches have been advocated for posterior teeth when only one or two tooth surfaces are missing. A recent study found no difference in the 3-year survival rate of ETT restored with a conservative composite restoration and those that received full-crown coverage when these teeth were missing one proximal surface and the proximal contact points were intact.^28^

The main drawback of conventional resin composite is that it undergoes shrinkage during polymerisation, which can lead to gap formation and microleakage.^17^ This results in more significant stresses accumulating within the tooth itself compared to the restoration, which may increase the risk of its fracture.^2,17^ New resin composites have been developed that can be applied in greater increment thickness (up to 4 mm) with reduced polymerisation shrinkage and stress accumulation. One such material, Smart dentin replacement material (SDR), is a new flowable bulk-fill composite, with low modulus of elasticity and high curing depth. Studies examining the fracture resistance of ETT restored with SDR have reported more favourable results^2,17^ vs conventional resin composite. Another dentin replacement material with physical properties (flexural strength and elastic modulus) similar to dentin is Biodentine. It is a tricalcium silicate-based bioactive restorative material used in vital pulp therapy.^15,18^

Biodentine and SDR restorations of ETT as alternatives to full-crown coverage have been previously proposed and examined on molars with only an access cavity preparation.^21^

This study aimed to compare fracture resistance of endodontically treated premolars with two missing surfaces (mesio-occlusal cavities) restored with either Biodentine or SDR, compared to a conservative, esthetic, but more expensive treatment modality, i.e. ceramic inlays. The null hypothesis was that there would be no difference between these materials in terms of resistance to fracture.

## Materials and Methods

This cross-sectional randomised controlled in-vitro study was conducted at Princess Nourah Bint Abdul Rahman University (PNU) Dental College simulation lab and King Saud University, Eng. Abdullah Bugshan Research Chair for Dental and Oral Rehabilitation lab. The study was exempted from ethical approval by PNU Institutional Review Board.

**Fig 1:**
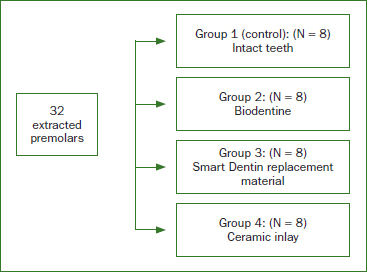
The four experimental groups.

Sample size calculation was performed using G*Power 3.1 software (Heinrich Heine University; Düsseldorf, Germany), estimating the power at 0.90 and a probability of Type 1 error α of 0.05. The sample size was set at 32 teeth to be divided into 4 groups. As such, 32 natural, sound premolars, extracted for periodontal or orthodontic reasons, were selected for this study. The criteria for tooth selection included: single, straight root canals; no visible caries, fractures or cracks on examination under the operating microscope (A3 series; Global, Surgical Corporation; St Louis, MO, USA); no signs of internal or external resorption or calcification; and a completely formed apex. Teeth with excessively short roots were also excluded. Preoperative radiographs were taken to confirm canal anatomy. Teeth were stored in saline solution before preparation and randomly divided into four equal experimental groups (n = 8), as described in Fig 1.

### Root Canal Preparation

For teeth in groups 2, 3 and 4, access cavities were prepared using endodontic access burs, after which the working length was determined with a size 10 K-file (Medin, A.S. Czech Republic). Canals were instrumented using Protaper Universal files (Dentsply Maillefer; Ballaigues, Switzerland) down to file F3.

According to the manufacturer’s instructions, all files were used on a 16:1 contra-angle handpiece attached to an electric motor (X-smart Endodontic Rotary Motor, Dentsply Sirona; Konstanz, Germany) at 350 rpm. Ethylenediaminetetraacetic acid (EDTA) 17% cream (MD-Chelcream Meta Biomed, Korea) was used as a chelating agent on the tip of each consecutive file. Canals were irrigated at each file change with 3 ml of 2.5% NaOCl using disposable plastic syringes. After instrumentation was completed, all canals received a final rinse of 5 ml saline. Canal filling was carried out using F3 gutta-percha cones (Dentsply Sirona) fitted to the working length, using the single cone technique with BC sealer. Canal orifices were covered with self-curing glass ionomer (SDI Riva; Bayswater, Victoria, Australia).

### Crown Mesio-Occlusal Cavity Preparation

Mesio-occlusal (MO) cavities were prepared in the crowns with standardised dimensions according to a method described by Bajunaid et al.^4^ Bucco-lingually, the proximal box extended in width to the intercuspal distance, while the gingival floor of the proximal box was 1 mm from the cementoenamel junction (CEJ). The occlusal isthmus width was set at half the intercuspal distance, with a depth of 1.5 mm. A distal marginal ridge of 1.5 mm was left intact. The axial wall of the proximal box was prepared at 60 to 90 degrees to the gingival floor with 6-degree divergence using a tapered diamond bur. All measurements were performed using a periodontal probe. Teeth were then restored with the material assigned for each group: group 2 teeth were restored with Biodentine (Septodont; Saint-Maur-des-Fossés, France), mixed according to the manufacturer’s instructions, then placed into the cavity with an amalgam carrier and adapted using a plugger and a plastic filling spatula. Several increments were required to fill the cavity. After 12 min, the hardness of the Biodentine was examined to confirm its setting. In group 3, cavities were cleaned and etched with 37% phosphoric acid (Total Etch, Ivoclar Vivadent; Schaan, Liechtenstein) for 15 s, and coated with bonding agent (Tetric N, Ivoclar Vivadent). Smart Dentin Replacement restorative material (SDR, Dentsply Sirona) was used to restore the cavities. All materials were applied according to the respective manufacturer’s instructions. For group 4, indirect ceramic inlay restorations were cemented using self-adhesive resin cement (Calibira Universal, Dentsply Sirona), following the manufacturer’s instructions. Table 1 shows the specifications of the materials used.

**Table 1:** Materials used in this study and their composition

Brand	Manufacturer	Lot No.	Preparation	Composition
Biodentine	Septodont; Saint-Maur-des-Fossés, France	B25878	Capsule containing powder and liquid mixed. Liquid: contains calcium chloride as an accelerator and a water-reducing agent.	Powder: tricalcium silicate, dicalcium silicate, calcium carbonate and oxide filler, iron oxide shade, and zirconium oxide. Liquid: contains calcium chloride as an accelerator and a water reducing agent.
Smart Dentine Replacement Material (SDR)	Dentsply Sirona; Konstanz, Germany	00050614	Flowable, light cured	Barium aluminofluoroborosilicate glass, strontium aluminofluorosilicate glass, modified urethane dimethacrylate resin, ethoxylated bisphenol A dimethacrylate (EBPADMA), triethylene glycol dimethacrylate (TEG-DMA), camphorquinone photoinitiator, butylated hydroxytoluene (BHT), UV stabiliser, titanium dioxide, and iron oxide pigments.
IPS E.Max CAD full-contour ceramic inlays	Ivoclar Vivadent; Schaan, Liechtenstein	N/A	Prepared for CAD/CAM use	Lithium disilicate (2SiO_2_–Li_2_O) dental ceramics. Partially crystalised blocks in a ‘blue state’ composed of various formulations of glass (namely SiO_2_, Li_2_O, P_2_O_5_, ZrO_2_, ZnO, K_2_O, and Al_2_O_3_ plus additional colorant ions) using glass technology via pressure casting.

All teeth were stored at 37°C in 100% humidity for 72 h following obturation and restoration.

### Weighing of Teeth

All teeth were weighed using a microbalance (Precisa EP225SM-DR; Marston Mills, MA, USA) after the endodontic and cavity preparation but before obturation. The results were analysed using one-way ANOVA, and no significant difference was found between the groups (p = 0.993).

### Measuring Compressive Load to Fracture and Mode of Fracture

**Fig 2:**
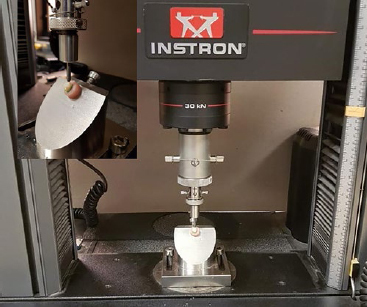
Compressive load testing set-up on the computer-controlled universal testing machine (Instron 8967).

After restoration and before load-resistance testing, all teeth were stored at 37°C in 100% humidity for 72 h post-restoration. Each tooth was mounted individually with its root embedded in a cylindrical mold, supported with light-body polyvinyl siloxane to simulate the periodontal ligament, and secured with self-curing acrylic resin up to 1 mm below the CEJ. The blocks were mounted individually on a special fixture on a computer-controlled universal testing machine (Instron 8967; Norwood, MA, USA). An axial compressive load was applied to the top palatal cusp at an angle of 45 degrees to its longitudinal axis with an oblique, steel compressive head. The rate of compressive loading was 2 mm/min until failure (fracture). The force at fracture was measured in Newtons (N) (Fig 2).

Specimens were then evaluated under a digital microscope with a magnification of 40X (Nikon SMZ1000 stereo zoom microscope) to define the fracture mode. A restorable fracture above the (CEJ) was considered favourable, and a non-restorable fracture below the CEJ unfavourable.

### Statistical Data Analysis

Data were analysed using SPSS (IBM SPSS Statistics for Windows, Version 22.0; Armonk, NY, USA) statistical software. Descriptive statistics were obtained, and one-way ANOVA followed by Tukey’s tests were used to compare the mean loads to failure between the four groups. The mode of fracture was compared using the Fisher’s exact test. Statistical significance was set at p˂0.05.

## Results

**Fig 3:**
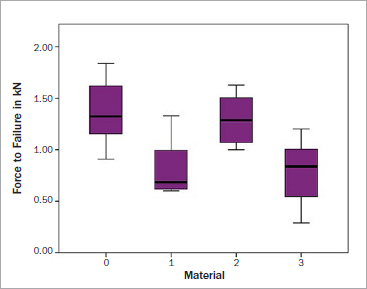
Maximum loads at failure and their mean values in the 4 study groups.

The maximum loads to failure for each group are shown in Fig 3. The control group showed the highest maximum loads to failure (1.368 kN ± 0.311). Multiple comparisons of the mean values showed a statistically significant difference between the groups (Table 2). Loads to failure were statistically significantly higher for teeth restored with Biodentine (1.293 kN ± 0.251) compared to those restored with either a ceramic inlay or SDR (p = 0.007; p = 0.012) (Table 3). However, there was no statistically significant difference between the Biodentine group and the control group (p = 0.952). While the lowest mean load to failure was recorded for the inlay group (0.782kN ± 0.309), it was not statistically significantly different when compared to the SDR group (p = 0.995).

**Table 2:** One-way ANOVA comparing the mean values of maximum loads to failure

Material	N	Mean	Std. Deviation	Std. Error	Sum of Squares	df	Mean Square	Sig
Control	8	1.3688	0.31128	0.11006	2.290	3	.763	*˂.0001
SDR	8	0.8162	0.26371	0.09323				
Biodentine	8	1.2938	0.25196	0.08908				
Inlay	8	0.7825	0.30918	0.10931				

*The mean difference is statistically significant at the 0.05 level. SDR: Smart dentin replacement.

**Table 3:** Post-hoc Tukey’s test comparing the mean values of maximum loads to failure between the groups

Material		Mean Difference	Std. Error	Sig.
Control	SDR	0.55250*	0.14264	0.003
	Biodentine	0.07500	0.14264	0.952
	Inlay	0.58625*	0.14264	0.002
SDR	Control	-0.55250*	0.14264	0.003
	Biodentine	-0.47750*	0.14264	0.012
	Inlay	0.03375	0.14264	0.995
Biodentine	Control	-0.07500	0.14264	0.952
	SDR	0.47750*	0.14264	0.012
	Inlay	0.51125*	0.14264	0.007
Inlay	Control	-0.58625*	0.14264	0.002
	SDR	-0.03375	0.14264	0.995
	Biodentine	-0.51125*	0.14264	0.007

*The mean difference is statistically significant at the 0.05 level. SDR: Smart dentin replacement.

**Fig 4:**
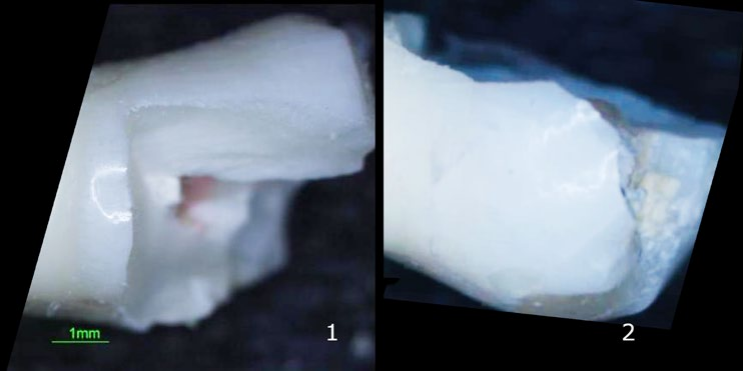
Mode of fracture: 1. control group; 2. Biodentine group.

Although most teeth in the control group fractured above the CEJ, no statistically significant difference was found between the four groups concerning fracture line extension (Table 4) (Fig 4).

**Table 4:** Distribution and comparison of fracture line extension among the 4 groups

Material	Fracture Line Extension				
	Below CEJ	Above CEJ	Total	Fisher’s Exact Test	Exact Sig. (2-sided)
Sound tooth SDR Biodentine Inlay	2 (25%)	6 (75%)	8 (100%)	3.296	0.440
	5 (62.5%)	3 (37.5%)	8 (100%)		
	5 (62.5%)	3 (37.5%)	8 (100%)		
	5 (62.5%)	3 (37.5%)	8 (100%)		
Total	17	15	32		

The mean difference is statistically significant at the 0.05 level. SDR: Smart dentin replacement, CEJ: cementoenamel junction.

## Discussion

Access cavity preparation is an impairment to tooth fracture resistance.^9^ Accordingly, full-coverage restoration of ETT has been advocated for cuspal protection.^25^ Recent evidence proved that that survival rates of ETT with limited (one or two surfaces) loss of coronal tooth structure are similar, whether restored with full-coverage restorations or with direct composite restorations.^28^

The purpose of this study was to explore alternatives to complete-coverage restorations of ETT with tooth structure loss due to class II deep dentinal caries. In the present study, teeth restored with Biodentine showed resistance to fracture comparable to that of sound teeth and statistically significantly higher than those restored with either SDR or ceramic inlays. This is an exciting finding, mirrored in other studies,^5^ showing that this material may have the capacity to compensate for the loss of marginal ridges that impact fracture resistance of premolars.^27^

Biodentine is a calcium silicate-based material that has was introduced for vital pulp therapy. It is considered a dentin substitute with compressive strength, elastic modulus, and microhardness similar to that of natural dentin.^8^ The product sheet of Biodentine describes one of its features as the ability to continue improving compressive strength over time, until reaching a range similar to that of natural dentin. Biodentine was reported to have the highest compressive strength compared to the other Bio aggregate and intermediate restorative materials.^15,22^

Although Biodentine is not advocated as a final restorative material, a study by Koubi et al^18^ proposed its use for posterior restorations, with favourable surface properties such as good marginal adaptation up to six months. This good marginal adaptation is due to the ability of Biodentine’s calcium silicate to form hydroxyapatite crystals at the surface. These crystals have the potential to increase the sealing ability. Another argument for using Biodentine is that it does not require any specific preparation of the dentin walls, which would conserve more tooth structure.^22^

After six months, however, abrasion was detected in the Biodentine restorations.^18^ Therefore, it was recommended to add a layer of direct composite after waiting more than two weeks to allow the Biodentine to undergo sufficient maturation to withstand contraction forces from the composite. This implies that further study is required to observe how adding such a layer would affect the resistance of ETT to fracture.

A study by Hiremath et al^16^ found that endodontically treated teeth with class I cavities restored using Biodentine showed the lowest resistance to fracture compared to composite and fiber-reinforced composite. This contradiction the results of the present study. There is no definitive explanation for this discrepancy; however, in the former study, it was not evident where the forces of the universal testing machine contacted the tooth’s occlusal surface, which may have influenced the result. Altering the loading position in addition to the inclination of the cusp itself was found to influence the stresses reproduced within the tooth and thus its fracture resistance.^20^

Polymerisation shrinkage causing marginal gap formation and microleakage is the chief disadvantage of conventional composite resin restorations.^17^ This polymerisation shrinkage leads to greater stress concentration within the tooth itself compared to the restoration, increasing the risk of tooth fracture.^2,17^ Although SDR undergoes reduced polymerisation shrinkage and stress accumulation, restoring ETT with SDR in the present study resulted in statistically significantly less resistance to fracture compared to both Biodentine and the sound control teeth. In contrast, Atalay et al^2^ reported no difference in terms of resistance to fracture between teeth restored with SDR and those with other types of nanohybrid and posterior composite resins. Many studies have reported that SDR showed less fracture resistance than other posterior restorative materials.^10,13^ This was attributed to its low filler loading.^19^

A meta-analysis study on survival rates of teeth restored with inlays reported long-term survival after 5 to 10 years. Tooth vitality was an influential factor, with inlays surviving longer on vital teeth. However, the most frequent cause of failure of these restorations was a fracture.

Not only are ceramic inlays the most expensive and time-consuming restoration in this study, they also exhibited the lowest fracture resistance. This is in agreement with previous studies that reported higher failure rates for all-ceramic restorations performed on endodontically treated teeth.^6,12^ This limits the indication for such restorations to vital teeth that are not subject to heavy occlusal loading.^6^

The results of this study are in accordance with those of Mergulhão et al,^23^ who reported no statistically significant difference between bulk-fill composite restorations and ceramic inlays in terms of fracture resistance. The latter study also found that teeth restored with these materials were not statistically significantly different from sound teeth. This was not the case in the present study. Teeth restored with SDR and ceramic inlays were statistically significantly less resistant to fracture than intact teeth. A study by Bajunaid et al^4^ showed that teeth restored with ceramic inlays were statistically significantly less resistant to tooth fracture compared to those restored with direct composite restorations.

Most studies showed that intact teeth were more likely to fracture in a reparable manner. The present findings indicate no difference between the groups in terms of modes of fracture, while other studies performed on ETT with more statistically significant coronal structure loss (mesio-occluso-disto cavities) reported that composite restorations were more often associated with irreparable fractures.^7^ A previous study^4^ also done on ETT with only mesio-occlusal cavities found no statistically significant difference between composite and ceramic inlays in terms of modes of failure.

While the ability to simulate the actual clinical/oral setting is limited in an in-vitro study such as this one, making it difficult to extrapolate the results directly to a clinical situation, it provides a strong starting point from which future randomised clinical investigations can be conducted.

Finally, the main limitation of the present study is its static loading design. It does not simulate the complexity of factors to which teeth are subjected in the oral cavity, especially the cyclic nature of the chewing forces. Due to their fluctuating nature, these forces can lead to failure at a much lower forces than those caused by static loading.^1^ The study also did not subject the tested materials to aging processes that simulate the complex environmental stresses (e.g. thermal) they must withstand in the oral cavity. Therefore, it is recommended that further investigations employing cyclic loading and thermal cycling be carried out to corroborate the current findings.

## Conclusion

In this in-vitro study, endodontically treated premolars with mesial-occlusal cavity preparations restored with Biodentine were more resistant to fracture than those restored with either SDR or ceramic inlays. Biodentine may prove a promising material to restore endodontically treated teeth with two missing surfaces, while ceramic inlays are not recommended.
